# Supplementary material for: Strong Aversive Conditioning Triggers a Long-Lasting Generalized Aversion
Source: Front Cell Neurosci. 2022 Feb 28;16:854315. doi: 10.3389/fncel.2022.854315 (PMC8918528; doi:10.3389/fncel.2022.854315)
Supplement: Supplementary file 1 [file Data_Sheet_1.PDF]

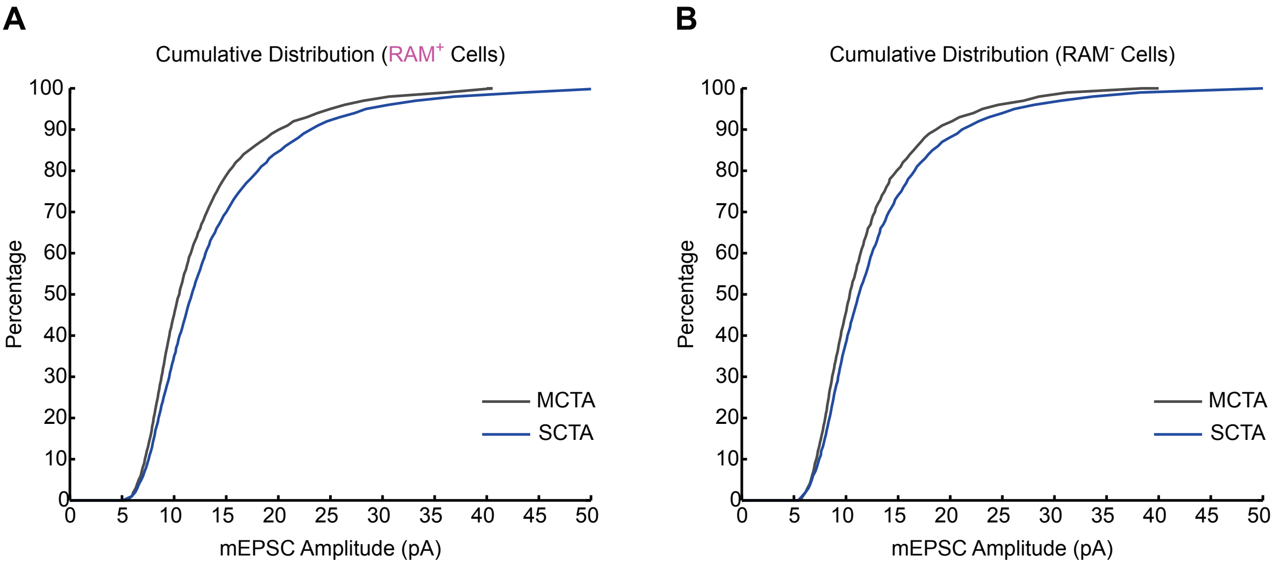

Supplementary Figure 1. Cumulative histograms of mEPSC amplitudes pertaining to **Figure 3** in the manuscript. (**A**) Cumulative histogram of mEPSC amplitudes sampled from RAM+ neurons following moderate or strong CTA conditioning (two-sample Kolmogorov-Smirnov test, $p = 0.0012$). (**B**) Cumulative histogram of mEPSC amplitudes sampled from RAM- neurons following moderate or strong CTA conditioning (two-sample Kolmogorov-Smirnov test, $p = 0.0010$).

Supp. Figure 2

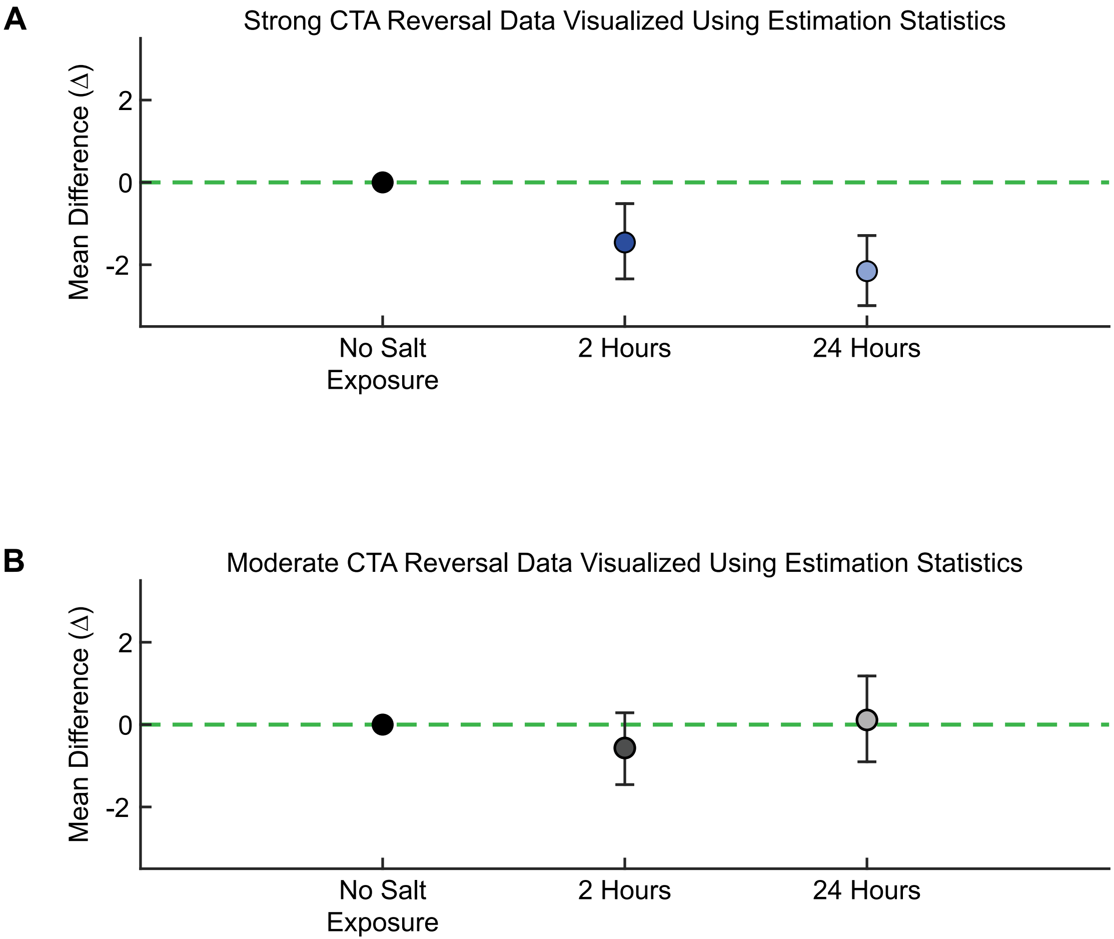

Supplementary Figure 2. Data pertaining to **Figure 4** of the manuscript visualized using modified Cumming estimation plot. For both (**A**) and (**B**), the mean difference for 2 comparisons against a shared control “No Salt Exposure” are shown above. The absolute values are plotted in the main body of the manuscript. Here, each mean difference is depicted as a dot. Each 95% confidence interval is indicated by the ends of the vertical error bars. This plot was generated using DABEST for MATLAB (Ho et al., 2019).

Supp. Figure 3

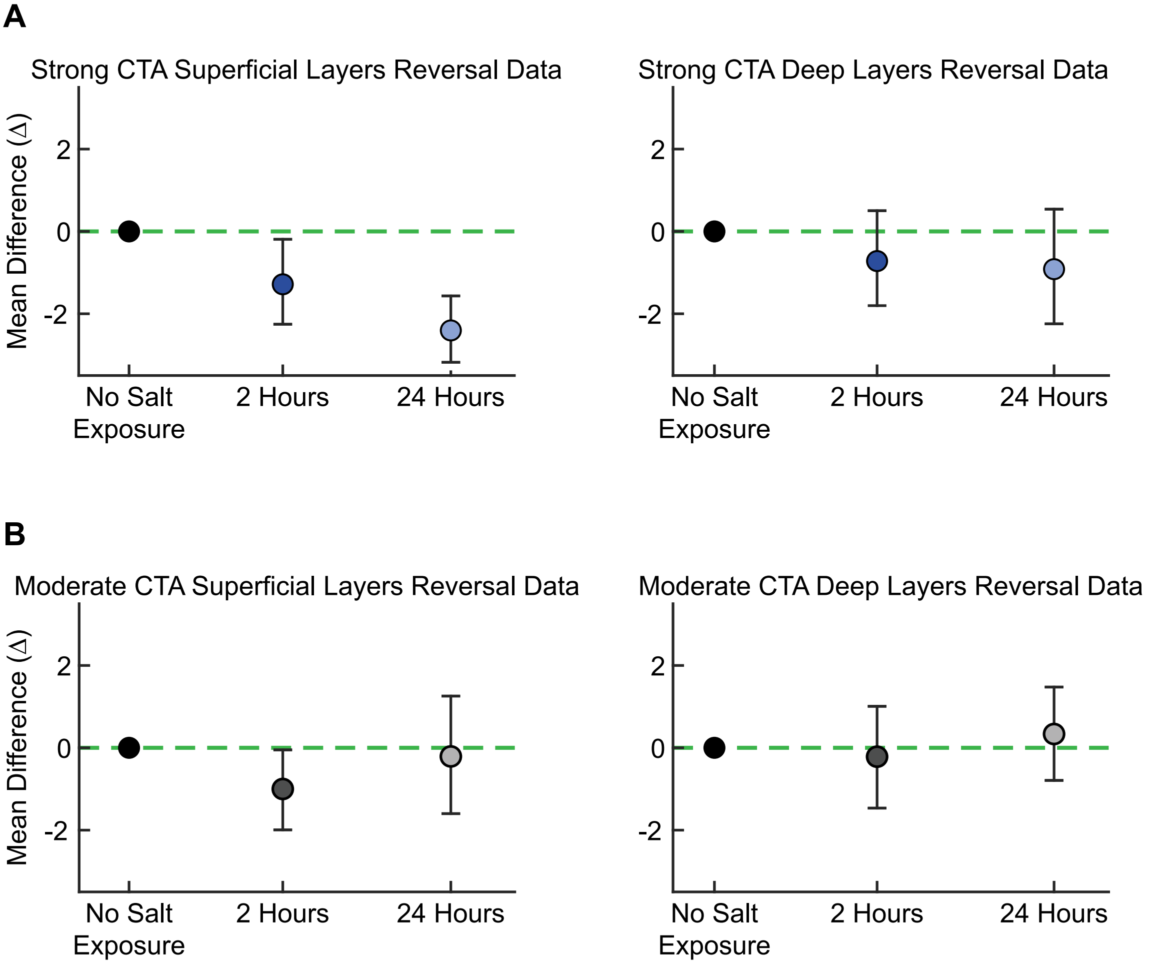

Supplementary Figure 3. Data pertaining to **Figure 5** of the manuscript visualized using modified Cumming estimation plot. For both (**A**) and (**B**), the mean difference for 2 comparisons against a shared control "No Salt Exposure" are shown above for data collected from superficial layers (left panels) and data collected from deep layers (right panels). The absolute values are plotted in the main body of the manuscript. Here, each mean difference is depicted as a dot. Each 95% confidence interval is indicated by the ends of the vertical error bars. This plot was generated using DABEST for MATLAB (Ho et al., 2019).

Supp. Figure 4

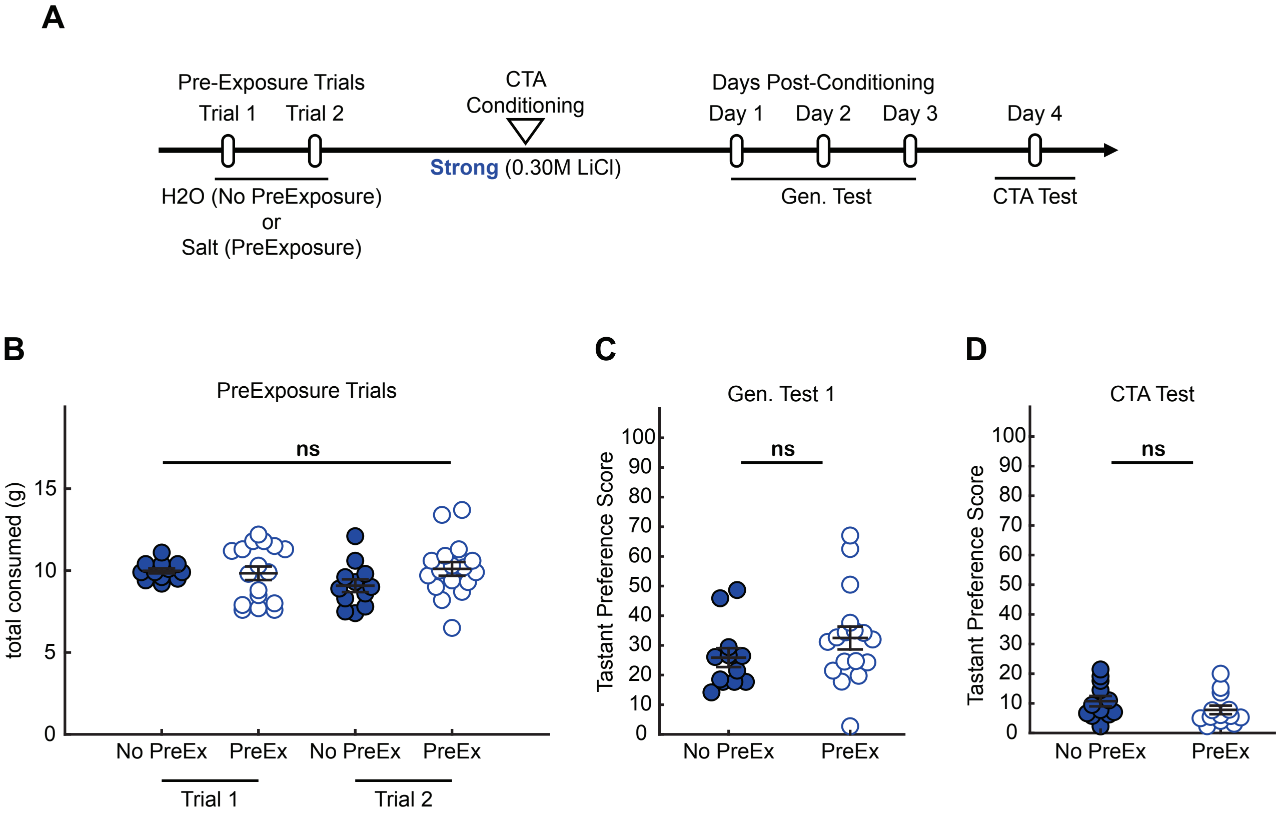

Supplementary Figure 4. Experiments evaluating the role of taste novelty in strong CTA induced generalized aversion. (**A**) PreExposure-generalized aversion behavioral paradigm. Animals were preexposed to salt (PreEx), or given water as a control (No PreEx). (**B**) Total consumption during PreExposure trials. There were no differences in total consumption between animals given salt or water (One-way ANOVA, $p = 0.2970$). (**C**) Gen. Test following strong CTA conditioning. PreExposure to the generalized tastant does significantly attenuate the generalized aversion (two-sample t test, $p = 0.2239$). (**D**) CTA Test, preference score for saccharin. All animals demonstrated similar levels of aversion to the conditioned stimulus (two-sample t test, $p = 0.2071$).
